# The Effect of Self Care Education Based on Orem’s Nursing Theory on Quality of Life and Self-Efficacy in Patients with Hypertension: A Quasi-Experimental Study

**DOI:** 10.30476/IJCBNM.2020.81690.0

**Published:** 2020-04

**Authors:** Zahra Khademian, Farzaneh Kazemi Ara, Sakineh Gholamzadeh

**Affiliations:** 1 Department of Nursing, School of Nursing and Midwifery, Shiraz University of Medical Sciences, Shiraz, Iran; 2 Community Based Psychiatric Care Research Center, Department of Nursing, School of Nursing and Midwifery, Shiraz University of Medical Sciences, Shiraz, Iran

**Keywords:** Hypertension, Nursing theory, Orem’s self-care deficit nursing theory, Quality of life, Self-care, Self-efficacy

## Abstract

**Background:**

Improvement of the quality of life and self-efficacy of patients with hypertension is essential. The present study aimed to determine the effect of self-care education based on Orem’s nursing theory on the quality of life and self-efficacy in patients with hypertension.

**Methods:**

This quasi-experimental study was conducted in Mamasani, Iran, 2015. Eighty patients were selected using convenient sampling and divided equally into two control and experimental groups based on random allocation. An educational program based on Orem’s nursing theory and according to the needs of patients was conducted in the experimental group. Data were collected before, immediately after, and eight weeks after the intervention using “Quality of Life of Cardiac Patients” and “Strategies Used by People to Promote Health” Questionnaires. Data were analyzed using SPSS 18; Chi-square test, independent t-test, and Analysis of Variances with Repeated Measures were used to analyze the data.

**Results:**

The mean score of the quality of life in the experimental group was significantly higher than the control group eight weeks after the intervention (106.5±26.5 vs. 85.5±22.5, P=0.03). However, this difference was not significant immediately after the intervention (94.4±25.3 vs. 87.2±22.8, P=0.32). The mean scores of self-efficacy were not significantly different from those of the control group immediately after (68.5±12.7 vs. 66.5±12.2, P=0.47) and eight weeks after the intervention (70.5±13.5 vs. 65.7±12.0, P=0.10).

**Conclusion:**

The results showed that training self-care based on Orem’s theory can improve the quality of life of patients with hypertension. Therefore, it is recommended that nurses in outpatient care of patients with hypertension should apply this theory.

**Trial Registration Number:** IRCT2015081323606N1.

## INTRODUCTION

Hypertension is one of the most common cardiovascular diseases in developed and developing countries. ^1^ The prevalence of hypertension among adults in the United States was reported 29.1%. ^2^ In Iran, the prevalence of hypertension was reported 17.3%. ^3^

Hypertension has an obvious impact on people’s health and social relations, and increases mortality. ^3 , 4^ Therefore, careful attention to appropriate treatment is essential. Evidence indicates that the United States and Canada have been successful in achieving optimal control of the disease in 53% and 66% of cases, respectively. ^5^ Also, 50.2% of Iranian patients with hypertension had controlled blood pressure. ^6^

Chronic illnesses threaten not only the physical health of patients, but also their mental and social health. Therefore, measuring the quality of life, especially in chronic diseases, is of particular importance. Quality of life is identified as one of the main indicators of cardiovascular health. Additionally, it is considered as an important measure of treatment outcome. ^7^ Evidence shows that the quality of life in patients with hypertension is poor and it is less than the normotensive people. ^7 - 10^ Therefore, in order to improve the health status and treatment outcome in patients with hypertension, it is necessary to find appropriate interventions to improve their quality of life. Previous studies have investigated the effectiveness of psychological interventions, ^11^ application of nursing theories, ^12 - 14^ and integrated care interventions (e.g. case management, multidisciplinary teams, discharge management) ^15^ on improving the quality of life in patients with chronic conditions. 

Hypertension treatment may continue throughout life, but some patients ignore its treatment; that is why they need to have regular follow-ups. ^16^ Self-efficacy is one of the main structures in Bandura’s social cognitive theory and it refers to the assurance of an individual about his ability to perform certain behaviors. ^17^ It is believed that the sense of self-efficacy and related behaviors is the key to successful treatment, increased self-care behaviors, and improved quality of life. ^18 - 20^ A previous study showed that elderly men with hypertension had unsatisfactory self-efficacy and a self-management program improved their lifestyle as to sodium intake, physical activity, fruit and vegetable consumption and weight loss. ^21^ In addition, self-efficacy was significantly associated with adherence to most of the hypertension self-care behaviors. ^20^ Therefore, it is necessary to find effective ways to improve self-efficacy among patients with hypertension. Previous studies have shown the effectiveness of self-management, ^22 , 23^ health promotion strategies education, ^18^ and application of nursing theories and models ^24 - 26^ on the self-efficacy of patients with chronic diseases. 

Given the chronic nature of hypertension and the likelihood of impacting the quality of life and self-efficacy in patients, it is necessary to conduct researches which aim at assessing the effectiveness of various methods of improving the quality of life and self-efficacy among these patients. Considering the important role of nurses in this field, ^27^ it is necessary to examine the effect of nursing theories on strengthening self-efficacy in patients with hypertension and improving their quality of life. The use of nursing theories is proposed to provide patient-centered care. ^28^ Furthermore, patients with chronic illnesses need motivation, experience, and skill to perform the behaviors that are needed to maintain and improve their health and quality of life, all of which being rooted in the concept of self-care. ^29^ Orem’s nursing theory mainly focuses on this concept. Therefore, it can provide an appropriate framework for studies on patients with chronic illness. 

Based on Orem’s nursing theory, self-care is considered as activities that people engage in to maintain, restore or improve their health. Nurses do not consider patients as inactive and mere recipients of health services; rather, they consider patients as strong, reliable, responsible, and capable of decision-making who can take care of their health appropriately. Orem defined three nursing systems including wholly compensatory, partially compensatory, and supportive-educative systems. The nurse’s roles in the supportive educational system are taken when the patient is ready to learn something, but he/she cannot do it without help and guidance. ^30^

The effectiveness of Orem’s theory on the quality of life ^12 , 13 , 31^ and self-efficacy ^24^ of patients with chronic diseases has been investigated. However, sufficient research has not been conducted on the effectiveness of this theory on the quality of life and self-efficacy of patients with hypertension. In addition, few interventions have been performed in terms of self-efficacy and quality of life in these patients. Therefore, the present study aimed to determine the effect of self-care education based on Orem’s nursing theory on the quality of life and self-efficacy in patients with hypertension. 

## MATERIALS AND METHODS

This is a quasi-experimental study which was conducted between August to November, 2015 in Valie-Asr hospital outpatient unit in Mamasani, Fars, Iran. All eligible patients admitted to the hypertension outpatient clinic during a one-month period were selected through convenience sampling. 

The sample size was calculated based on data from a pilot study and by α=0.05, β=0.20, s=0.48, and (µ_1_-µ_2_)=0.3, the minimum sample size was 80.

$N=\frac{2{\left(Z_{1-\alpha /2}+Z_{1-\beta }\right)}^{2}2\sigma^{2}}{{\left(\mu_{1}-\mu_{2}\right)}^{2}}$

Considering the possibility of 10% attrition, when the sample size reached 88, sampling was stopped. Then, they were assigned equally into two experimental and control groups by simple randomization, using the table of random numbers and sampling frame (list of patients available in the clinic). Four patients in experimental group did not continue the study due to lack of willingness, long distance from their place of residence and poor health status; also, four patients in the control group did not return to the clinic to complete the post-test because of the long distance from their place of residence. Eventually, 40 patients in the experimental group and 40 in the control group completed the study.

Inclusion criteria included being registered as patients with hypertension and having medical record in the clinic, a history of at least one anti-hypertension drug for a year, age between 18-55 years, minimum education (elementary), the ability to understand and communicate in the Persian language, and willingness to participate in the study. The people of Mamasani mainly speak with Lori accent. Therefore, given other communication problems of older adults ^32^ audio-speaking Farsi for clients older than 55 years was difficult. Therefore, the maximum age of 55 was considered as the inclusion criteria. Exclusion criteria included known mental disorders, drug addiction in the previous year, history of major cardiac surgery within the last 6 months, prolonged hospitalization from two years ago, and studying in medicine-related majors.

Research instruments included “Quality of Life of Cardiac Patients” and “Strategies Used by People to Promote Health” (SUPPH-29) questionnaires. In addition, demographic information such as age, sex, marital status, education, occupation, and place of residence were asked via interviews and recorded in a specific form.

The Quality of Life of Cardiac Patients’ Questionnaire is the modified and completed version of quality of life after heart attack which was designed by Lim et al. (1993) in English ^33^ and edited by Valenti et al. (1996). ^34^ The questionnaire assesses the effects of cardiovascular disease and its treatment on the patient’s physical, emotional and social activities. This tool consists of 27 items. Each item has a seven-point Likert-type response rating from never (score=1) to always (score=7). The scores range from 27 to 189 and higher scores indicate better quality of life. Lim et al. performed factor analysis using varimax rotation and three factors were emerged that explained 61% of the total variation. ^33^ Furthermore, Valenti et al. used principal components factor analysis with a varimax rotation and showed that the modified questionnaire increased the total variance explained by the original factors from 65.8% to 66.5%. In addition, they ensured internal consistencies by Cronbach’s alpha coefficient of 0.95 for social and emotional dimensions, and 0.93 for physical dimension. ^34^ Asadi-Lari et al. ensured the construct validity of the Persian version of the questionnaire in Iran by principal component analysis. They showed that the instrument’s three factors explained 63% of the total variation. Furthermore, they confirmed its content validity using backward-forward method and its reliability with Cronbach’s Alpha of 0.95 for global score, 0.92 for the emotional and physical domains, and 0.94 for the social domain. ^35^

The SUPPH-29 is a self-report questionnaire designed for the first time by Lev et al. (1996) to measure self-efficacy in self-care. ^36^ The questionnaire has 29 items that are answered based on a five point Likert-type scale from very low (score=1) to very high (score=5). The score ranges from 29 to 145. Scores above 90 show high self-efficacy, 67-90 average self-efficacy, and less than 67 low self-efficacy. Lev et al. performed exploratory factor analysis with direct quartimin oblique on 29 items and four factors emerged. These factors were named coping, stress reduction, decision making, and enjoying life and explained 81% of the total variance. Lev et al. also explored convergent and discriminant validity of the instrument and showed its correlation with Health Behavior Scale at 0.61 (P<0.001) and with the Revised Grief Experience Inventory at -0.38 (P<0.01). They concluded that the higher self-efficacy of the respondents, the more positive their health beliefs were and the less grief they experienced. The researchers also reported the SUPPH test-retest stability coefficient of 0.94. ^36^ Moattari et al. translated the instrument into Persian and then back-translated it into English. Then, the experts confirmed the content validity of the Persian version of the instrument. The overall reliability was ensured by Cronbach’s alpha coefficient of 0.91. ^37^ The construct validity of the Persian version of the questionnaire has not been determined in Iran.

Both the experimental and control groups completed the pre-test questionnaires; then the educational program based on Orem’s nursing theory was conducted in the experimental group. Based on this theory, the program has several unique features such as identifying universal and health deviation self-care requisites, and emphasizing the independence of individuals and their active participation in self-care. Universal self-care requisites are those that must be met in order to maintain a human’s structural and functional integrity. These include the need for maintenance of a sufficient intake of air, water, food and provision of care associated with elimination processes and excrements balance between, exercise and rest, balance between solitude and social interaction, risk prevention and promotion of functioning and development. Health deviation self-care requisites occur secondary to diseases, diagnosis or treatment. ^30^ In this study, firstly, needs assessment was done and the universal and health deviation self-care requisites were identified based on the theory. Then, one of the researchers designed the self-care program according to the identified self-care requisites based on Orem’s supportive educational nursing system. The educational program was certified and confirmed by a cardiologist. Afterwards, four 45-minute sessions of training were conducted on a weekly basis over a month for individuals in the experimental group. The first three sessions were individual and the fourth was in group. At the end of each session, a booklet related to the training of the given session was distributed among the experimental group. Immediately after the fourth session, two questionnaires were administered. During the next eight weeks, two telephone follow-ups were done to assess the implementation of the trainings provided to the patients, answer their questions, and encourage them to actively participate in self-care activities. In addition, the researcher’s phone number was given to the patients in case they needed to contact the researcher. 

During the study period, a routine intervention was conducted in the control group that included monthly visits by a physician and blood pressure assessment. The intervention group received the routine intervention, in addition to the above-mentioned intervention. After these eight weeks of the intervention, the questionnaires were completed again by patients in the experimental and control groups. Because the intervention was educational and the control group did not receive any special intervention, the patients were aware of their assigned group. Therefore, there was no possibility of blindness.

Data analysis was done by SPSS software version 18.0. Descriptive statistics were used to describe the variables. To compare the two groups in terms of qualitative and quantitative demographic characteristics, we used Chi-square test, and t-test. The overall quality of life and self-efficacy score before, immediately after and eight weeks after the intervention in each group was separately compared by performing repeated measures analysis of variances. The reason for performing this test separately in each group was that in the initial analysis, the effect of time-group interaction for the quality of life (P<0.001) and self-efficacy (P=0.03) was significant. Therefore, to compare the quality of life and self-efficacy in patients between the two groups before and after the intervention, we used independent t-test. P-value of <0.05 was considered statistically significant. 

The ethics Committee at Shiraz University of Medical Sciences (No. IR.SUMS.REC.1394.44) approved and the authorities of the hospital confirmed the study. The study objectives were explained to the patients and informed consent was obtained from them. 

## RESULTS

The sample consisted of 42 (52.5%) male and 38 (47.5%) female patients with hypertension with a mean age of 47.52±5.98 years. The majority of patients were married 43 (53.8%), had elementary education 36 (45%), were unemployed or retired 51 (63.8%) and were city dwellers 46 (57.5%). The mean age of the experimental (46.7±5.8) and control groups (48.3±6.08) was matched (P=0.23). In addition, the experimental and control groups were matched in terms of gender, marital status, education and employment status (Table 1).

**Table 1:** Demographic characteristics of patients with hypertension in the experimental and control groups

Demographic variables	Experimental group (N=40)	Control group (N=40)	P value*
	N (%)	N (%)	
Gender			
Male	22 (55)	20 (50)	0.96
Female	18 (45)	20 (50)	
Marital status			
Married	22 (55)	21 (52.50)	0.77
Single	10 (25)	9 (22.50)	
Widow	5 (12.50)	6 (15)	
Divorced	3 (7.50)	4 (10)	
Education			
Elementary	16 (40)	20 (50)	0.76
Diploma	13 (32.50)	11 (27.50)	
Associate degree	8 (20)	7 (17.50)	
Bachelor’s Degree or higher	3 (7.50)	2 (5)	
Employment status			
Unemployed	9 (22.50)	9 (22.50)	0.73
Retired	15 (37.50)	18 (45)	
Employed	16 (40)	13 (32.50)	
Place of residence			
City	24 (60)	22 (55)	0.68
Rural area	16 (40)	18 (45)	

* Chi-square test

The mean scores of the quality of life (87.02±24.3 vs. 86.3±23.3, P=0.92) and self-efficacy (65.5±12.3 vs. 65.3±12.1, P=0.92) in patients with hypertension in both groups were not significantly different before the intervention (Table 2). 

**Table 2:** The comparison of the mean scores of the quality of life and self-efficacy in patients with hypertension before, immediately after, and eight weeks after the intervention within and between the experimental and control groups

Time		Before the intervention	Immediately after the intervention	Eight weeks after the intervention	F	P value ** (Within group)
Statistic		Mean±SD	Mean±SD	Mean±SD		
	Group					
Quality of life	Experimental	87.02±24.30	94.40±25.30	106.50±26.50	8.33	<0.001
	Control	86.30±23.30	87.20±22.80	85.50±22.50	3.86	0.03
P value* (Between groups)	0.91	0.32	0.03		
Self-efficacy	Experimental	65.50±12.30	68.50±12.70	70.50±13.50	8.75	<0.001
	Control	65.30±12.10	66.50±12.20	65.70±12.00	4.80	0.01
P value*(Between groups)	0.92	0.47	0.10		

* Independent t-test;

** Repeated measurement ANOVA

The mean scores of the quality of life in the experimental and control groups were not significantly different immediately after the intervention (94.4±25.3 vs. 87.2±22.8, P=0.32). However, the mean score of the quality of life in the experimental group was significantly higher than the control group eight weeks after the intervention (106.5±26.5 vs. 85.5±22.5, P=0.03) (Table 2). 

Findings indicate that the mean scores of self-efficacy were somewhat higher in the experimental group than control group immediately (68.5±12.7 vs. 66.5±12.2, P=0.47) and eight weeks (70.5±13.5 vs. 65.7±12.0, P=0.10) after the intervention. However, these differences were not statistically significant (Table 2). 

## DISCUSSION

Based on the results of this study, the quality of life of the patients in the experimental group increased significantly eight weeks after the intervention. However, the differences in the self-efficacy mean scores between the two groups were not statistically significant. 

According to the findings of this study, designing and implementing Orem self-care educational program based on the needs of patients with hypertension along with follow-ups can be effective in improving the quality of life of these patients. The changes were significant after eight weeks of the intervention. Therefore, the role of follow-ups and the patients’ confidence on the nurse’s availability to answer their questions is important. Similar to these findings, previous studies showed that the self-care program had a positive effect on the quality of life in patients with migraine, ^12^ multiple sclerosis ^13^ and those undergoing hemodialysis. ^31^ Contrary to these findings, the self-care program was not effective on the quality of life in patients with sickle cell anemia. ^38^ The reason for lack of consistency may lie in the disease type, number of training sessions, and duration of the study.

The findings showed that the intervention had not a statistically positive effect on self-efficacy in patients with hypertension. These results are in the same line with those obtained by another study reported that the Orem self-care model was not effective on the self-efficacy of hemodialysis patients in Urmia, Iran. ^39^ On the other hand, in another study an educational program based on Orem’s model improved the self-efficacy of patients with multiple sclerosis. ^24^ In addition, the administration of a self-management program in patients with primary hypertension ^23^ and ulcerative colitis ^22^ led to improved self-efficacy. It should be noted that most of the patients in the current study had elementary education. This issue, in addition to the different nature of the diseases, may explain the differences of these findings with some previous studies. 

We expected more changes in self-efficacy scores after the intervention. Hence, it is recommended that in future studies the intervention should be performed over a longer period and with a higher frequency of training to allow examination of the effect of a long-term intervention on the improvement of self-efficacy. 

One of the limitations of this study was the lack of blinding due to the awareness of the researcher and participants about the experimental and control groups. In addition, the SUPPH-29 is commonly used in different languages and, as it was mentioned previously, the validity and reliability of the English version and the Persian version were determined. However, we did not find the report of the construct validity of the Persian version of the questionnaire. It is suggested that the construct validity of the Persian version of this instrument should be confirmed in future studies. Furthermore, the Quality of Life of Cardiac Patients questionnaire was mainly designed for patients after myocardial infarction and its validity and reliability for patients with hypertension have not been investigated.

## CONCLUSION

The results of this study showed that designing and implementing the Orem self-care program can increase the quality of life of patients with hypertension. Therefore, it is recommended that nurses in the outpatient care units should apply Orem self-care model for patients with hypertension and the role of nurses in these settings should be strengthened. Given that the self-efficacy in patients in the experimental group did not increase significantly compared to the control group, it is also recommended that in the further studies, the follow-up and intervention should be continued in a longer term, so that the effects of the intervention on self-efficacy can be examined longitudinally.
